# Antibiotic utilization patterns, direct antibiotic acquisition costs, and hospital transfer outcomes in a pediatric emergency care unit: a retrospective study from Northeastern Brazil

**DOI:** 10.1016/j.rcsop.2026.100871

**Published:** 2026-11-04

**Authors:** Cristiane Almeida Rocha, Eduard F. Valenzuela, Fernanda Warken Rosa, Aníbal de Freitas Santos Júnior, Roberto Rodrigues Bandeira Tosta Maciel

**Affiliations:** Universidade do Estado da Bahia (UNEB), Departamento de Ciências da Vida (DCV), Rua Silveira Martins, 2555 - Cabula, 41150-000 Salvador, BA, Brazil

**Keywords:** Antibiotics, Direct costs, Pediatric emergency care, Health economics, Antimicrobial stewardship

## Abstract

**Background:**

Antibiotic prescribing in pediatric emergency care represents an important clinical and economic challenge, particularly for antimicrobial stewardship and resource allocation. Evidence from pediatric emergency settings in low- and middle-income countries remains limited.

**Approach:**

This retrospective study evaluated antibiotic prescribing patterns, direct antibiotic acquisition costs, and hospital-transfer status in a pediatric emergency care unit in Northeastern Brazil from 2019 to 2021. The full prescription dataset was used for descriptive analyses and cumulative acquisition cost estimation, whereas complete-case subsets were used for antibiotic unit-cost comparisons and hospital-transfer analyses. Direct costs were estimated using institutional acquisition prices. Statistical analyses included group comparisons, principal component analysis (PCA), and hierarchical cluster analysis (HCA).

**Results:**

The descriptive sample comprised 10,489 antibiotic prescription records, with 324 treatment episodes included in the unit-cost analysis and 302 in the hospital-transfer analysis. The estimated minimum cumulative direct antibiotic acquisition cost for the overall prescription dataset was USD 13,044.67, with benzathine benzylpenicillin, ceftriaxone, amoxicillin, and azithromycin accounting for the highest cumulative costs. Antibiotic unit costs differed significantly across diagnostic groups (*p* = 0.022). Among treatment episodes included in the unit-cost analysis, 78.1% of antibiotics were classified as Access and 21.9% as Watch according to the World Health Organization (WHO) Access, Watch, and Reserve (AWaRe) classification, with no Reserve antibiotics identified. Exploratory PCA and HCA showed differences in therapeutic profiles across diagnostic groups. Higher antibiotic unit costs were observed among transferred episodes, although only seven episodes involved transfer, limiting the interpretation of this exploratory comparison.

**Conclusions:**

Antibiotic utilization and direct antibiotic acquisition costs varied across pediatric diagnoses, highlighting opportunities for antimicrobial stewardship and pharmaceutical resource optimization in pediatric emergency care.

## Graphical abstract

**Figure:**
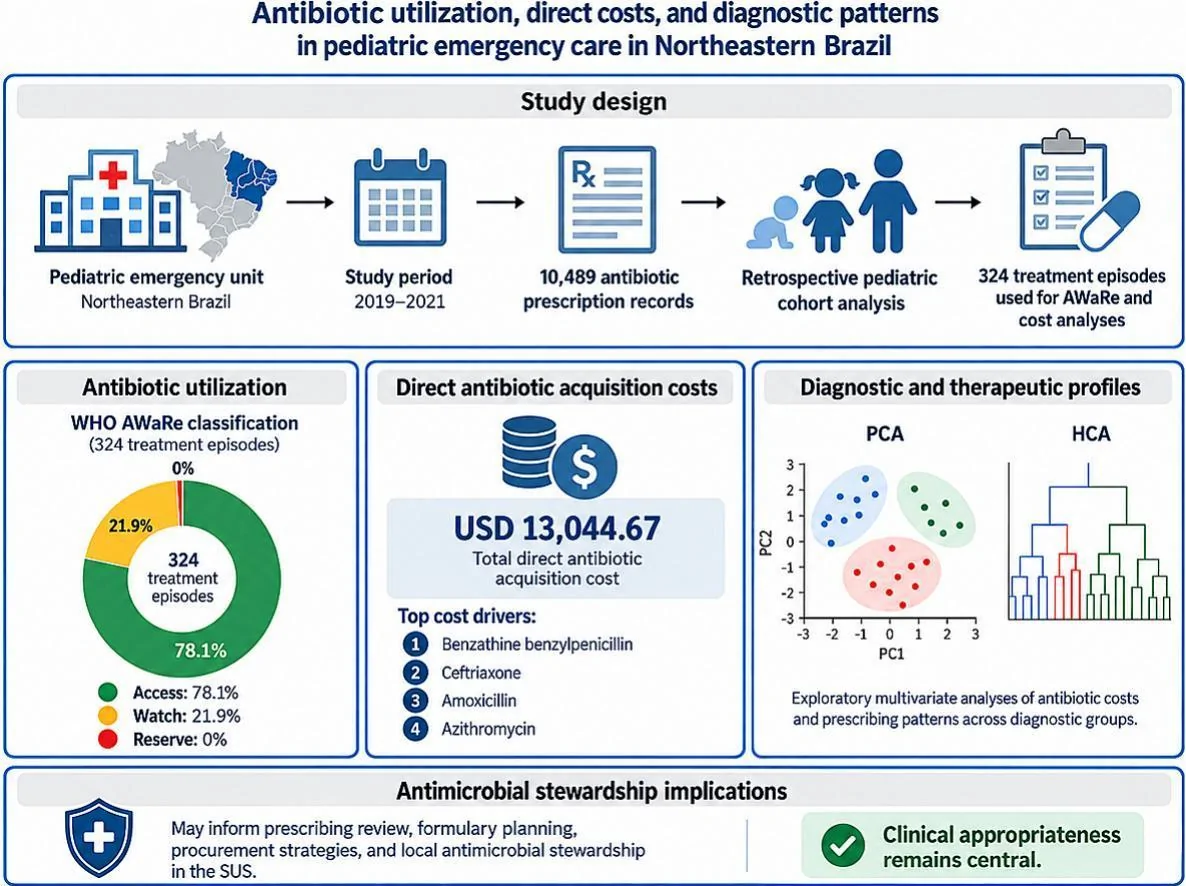

## 1 Introduction

Pediatric emergency care units play a central role in the management of acute infectious conditions, particularly among infants and young children, who are more vulnerable to respiratory, febrile, and gastrointestinal infections. In these settings, antibiotics are among the most frequently prescribed medications due to the high prevalence of suspected bacterial infections and the need for rapid clinical decision-making. However, empirical prescribing in urgent care environments may increase the risk of inappropriate antimicrobial use, especially in cases involving nonspecific diagnoses such as fever or acute respiratory symptoms.

The inappropriate use of antibiotics in pediatric care has emerged as a major public health concern due to its association with antimicrobial resistance, increased risk of adverse drug events, and avoidable healthcare expenditures.1., 2., 3. In recent years, antimicrobial stewardship programs have been increasingly recognized as essential strategies to optimize prescribing practices, improve clinical outcomes, and reduce unnecessary healthcare expenditures, particularly in pediatric settings. Recent international evidence has reinforced the importance of aligning antibiotic prescribing with evidence-based recommendations, such as the World Health Organization (WHO) Access, Watch, and Reserve (AWaRe) framework, to promote rational use and minimize inappropriate exposure to broad-spectrum agents.^4^ Recent WHO global surveillance reports further highlight the persistent burden of antimicrobial resistance and reinforce the need for systematic monitoring of antimicrobial use and resistance patterns.^5^ In pediatric acute and emergency care, stewardship recommendations emphasize evidence-based antibiotic selection and judicious use, particularly in clinical situations in which empirical treatment is frequently initiated.^6^ Likewise, structured antimicrobial stewardship programs have demonstrated potential to improve treatment efficiency and reduce costs associated with prolonged care and unnecessary hospital use.^7^

From a health economics perspective, antibiotic prescribing constitutes an important component of direct pharmaceutical expenditure in public healthcare services, and strategies aimed at optimizing antimicrobial use have demonstrated substantial potential to reduce healthcare costs and improve resource allocation efficiency.8., 9., 10. In Brazil, where the *Sistema Único de Saúde* (SUS) is responsible for delivering universal healthcare, understanding the economic impact of antimicrobial use is essential for the rational allocation of resources and the sustainability of emergency care services. Although several studies conducted in Brazil have evaluated antimicrobial use and associated costs in hospitalized pediatric populations, evidence specifically addressing pediatric emergency care units and intermediate outcomes of care remains scarce, particularly in medium-sized municipalities in Northeastern Brazil.11., 12., 13.

Therefore, this study aimed to evaluate antibiotic prescribing patterns, estimate direct antibiotic acquisition costs, and explore the relationship between antibiotic unit costs and hospital-transfer status as an intermediate outcome of care in a pediatric emergency care unit in Northeastern Brazil.

## 2 Methods

### 2.1 Study design and setting

A retrospective observational study was conducted from the perspective of the healthcare provider to evaluate direct antibiotic costs and hospital-transfer status as an intermediate outcome of care in a pediatric emergency care unit in Camaçari, Bahia, Northeastern Brazil. The study used routinely collected medical record data from children treated between January 2019 and December 2021 in a public emergency care service that operates as an intermediate level of care between primary healthcare and hospital admission.

### 2.2 Study population and eligibility criteria

The descriptive dataset comprised 10,489 antibiotic prescription records from pediatric patients who received at least one antimicrobial prescription during the study period. A single patient could contribute more than one treatment episode during the study period. Episodes separated by more than 30 days or corresponding to a newly documented acute clinical condition were considered independent episodes, whereas repeated visits or duplicate records related to the same clinical event were consolidated into a single treatment episode.

For the antibiotic unit-cost analysis, only treatment episodes with sufficient information to determine antibiotic acquisition costs were retained. Of the initial records, 8923 were excluded because of missing financial information or zero recorded medication costs, leaving 1566 prescription records with cost information. Zero-cost prescriptions were excluded from the economic analysis because they did not represent an identifiable acquisition cost to the healthcare service. An additional 1242 records were excluded because of incomplete or inconsistent information that prevented reliable identification and characterization of treatment episodes. After data cleaning, the analytical sample comprised 324 treatment episodes for the antibiotic unit-cost analysis. The complete analytical dataset used for the cost analyses is provided in the Supplementary Data (Cost_Analysis_Dataset worksheet).

Because hospital-transfer information was not available for all treatment episodes, 22 episodes were excluded from the hospital-transfer analysis, resulting in a final sample of 302 treatment episodes (295 non-transferred and 7 transferred). The sample-selection process and the analytical samples used at each stage are summarized in the STROBE-style flow diagram (Fig. 1).

**Fig. 1:**
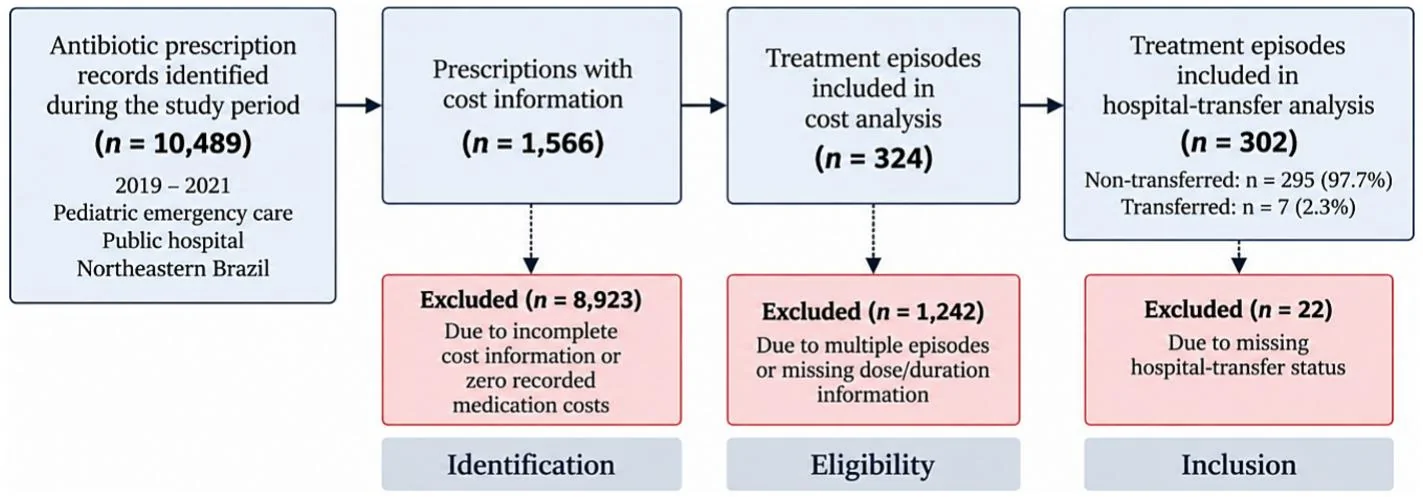
STROBE-style flow diagram of the selection of antibiotic prescription records and treatment episodes included in the cost and hospital-transfer outcome analyses.

### 2.3 Data sources and study variables

Data were obtained through a retrospective review of medical records. The extracted variables included age, sex, year of care, clinical diagnosis, prescribed antibiotics, and hospital transfer status. Clinical diagnoses were coded according to the International Classification of Diseases, 10th Revision (ICD-10), by trained personnel. Diagnostic coding was independently reviewed and validated by a second reviewer. Diagnoses were later grouped into aggregated clinical categories for comparative analyses. Antibiotic prescribing patterns were also described according to type of antimicrobial and calendar year. Treatment episodes involving antibiotic combinations were analyzed separately from single-drug prescriptions. The original study also consulted clinical references, including Micromedex, Red Book, and pediatric guidance documents, to contextualize prescribing patterns in relation to expected therapeutic use.

Antimicrobials were additionally categorized according to the WHO AWaRe classification to provide a descriptive assessment of prescribing patterns from an antimicrobial stewardship perspective. AWaRe categories were summarized as frequencies and percentages. Given the retrospective nature and limitations of the available data, the study did not assess the clinical appropriateness of antibiotic indication, dosing, treatment duration, or adherence to therapeutic guidelines. Therefore, the AWaRe analysis was interpreted strictly as a descriptive characterization of antimicrobial use rather than as an assessment of prescribing appropriateness.

### 2.4 Cost estimation

The economic analysis focused exclusively on direct antibiotic costs. Antibiotic unit cost was defined as the institutional acquisition price per unit of each antibiotic and was used for statistical comparisons across diagnostic groups and hospital-transfer categories (*n* = 324).

The cumulative direct antibiotic acquisition cost was estimated using the 10,489 prescription records. For each study year, the number of prescriptions for each antibiotic was multiplied by its corresponding unit acquisition price, assuming one unit per prescription. The resulting values were summed to estimate the minimum cumulative acquisition cost for 2019–2021.

Institutional acquisition prices were obtained separately for each study year (2019, 2020, and 2021) to account for annual variations in procurement costs. Costs originally recorded in Brazilian reais (R$) were converted to US dollars (USD) using the corresponding annual average R$/USD exchange rate published by the Central Bank of Brazil (2019: R$3.9451/US$1; 2020: R$5.1558/US$1; 2021: R$5.3950/US$1). No inflation adjustment or discounting was applied because of the short analytical time horizon. Costs are therefore reported as nominal USD. When no acquisition price was available for a specific year, the closest available price was used. For ceftriaxone, the average acquisition cost between intramuscular and intravenous formulations was adopted.

The cumulative estimate does not represent the actual cost of complete antibiotic treatments, as dosing, treatment duration, and the number of units administered were not considered.

### 2.5 Intermediate clinical outcome

Because primary clinical outcomes were incompletely recorded in the medical records, hospital transfer status was used as an intermediate outcome of care and was categorized as transfer or non-transfer. Non-transfer was not considered equivalent to clinical cure or treatment effectiveness. Hospital transfer may be influenced by factors beyond the patient's clinical response, including hospital capacity, physician decision-making, social circumstances, diagnostic uncertainty, and local referral protocols. Therefore, transfer status was interpreted as an intermediate outcome rather than a direct measure of antibiotic effectiveness or clinical resolution. Treatment episodes with missing hospital-transfer status (*n* = 22) were excluded from this specific analysis.

### 2.6 Statistical analysis

Descriptive analyses were performed for patient characteristics, antibiotic prescribing patterns, and direct antibiotic costs. Continuous cost variables were summarized using the mean and standard deviation (SD), together with the median and interquartile range (IQR), minimum, and maximum. No observations were excluded solely on the basis of extreme cost values, and the complete cost-analysis sample was retained for statistical analyses.

The distribution of antibiotic unit costs was assessed using histograms and Q–Q plots and formally evaluated using the Shapiro–Wilk test. Normality of residuals from the preliminary one-way analysis of variance (ANOVA) model was also assessed using the Shapiro–Wilk test and Q–Q plots, while homogeneity of variances across diagnostic groups was evaluated using Levene's test. Because antibiotic unit costs and ANOVA residuals showed significant departures from normality, non-parametric methods were used for the primary inferential analyses.

Differences in antibiotic unit costs across aggregated diagnostic groups were assessed using the Kruskal–Wallis test. When the overall test was significant, pairwise comparisons were performed using Dunn's post hoc test with Holm adjustment for multiple comparisons. Antibiotic unit costs between transferred and non-transferred treatment episodes were compared exploratorily using the Mann–Whitney *U* test (Wilcoxon rank-sum test), given the non-normal distribution, marked imbalance in group sizes, and limited number of transferred episodes. All tests were two-sided, with a significance level of 5%, and analyses were performed in R.

### 2.7 Exploratory multivariate analysis

To explore the relationship between aggregated diagnostic groups and therapeutic profiles, an exploratory principal component analysis (PCA) and hierarchical cluster analysis (HCA) were performed. Seven diagnostic groups (Fever syndrome, Other, Otitis, Respiratory infection, Skin infection, Tonsillitis/Pharyngitis, and urinary tract infection [UTI]) were included in the analyses. Because the multivariate analyses were conducted at the diagnostic-group level, the original treatment-episode-level dataset was aggregated by diagnostic group, and therapeutic indicators were expressed as percentages.

The final multivariate matrix comprised five therapeutic variables: parenteral administration, combination therapy, amoxicillin, benzathine benzylpenicillin, and ceftriaxone. Parenteral administration combined intramuscular and intravenous routes into a single indicator. Variables were centered and scaled prior to analysis. PCA was conducted in R software using the prcomp function. The number of components retained for interpretation was determined based on eigenvalues and cumulative explained variance. The first two principal components were the only components with eigenvalues >1 and jointly explained 93.89% of the total variance; therefore, they were retained for graphical and substantive interpretation.

To complement the PCA by examining similarities and dissimilarities among diagnostic-group therapeutic profiles, HCA was performed on the same standardized multivariate matrix using Euclidean distance and Ward's linkage method. The complete PCA loading matrix is provided in the Supplementary Data (PCA_HCA_Analysis worksheet; Table S6), and the aggregated therapeutic profiles used for both PCA and HCA are provided in Table S7 of the same worksheet.

## 3 Results

### 3.1 Patient characteristics and overall antibiotic use

A total of 10,489 antibiotic prescription records were included in the descriptive analysis. The demographic characteristics associated with these prescription records are summarized in Table 1. Prescription records for male patients accounted for 54.4% (*n* = 5707), while those for female patients represented 45.6% (*n* = 4782). The highest frequency of antibiotic prescriptions was observed among children aged 0–2 years, corresponding to 68.0% (*n* = 7132) of all prescription records. These findings indicate a substantial concentration of antibiotic use in early childhood.

**Table 1:** Demographic characteristics associated with pediatric antibiotic prescription records (2019–2021).

Variable	N	%
Total prescription records	10,489	100.0
Male	5707	54.4
Female	4782	45.6
Age 0–2 years	7132	68.0
Age 3–12 years	3357	32.0

### 3.2 Antibiotic prescribing patterns and temporal distribution

Among the antibiotics prescribed throughout the study period, azithromycin, benzathine benzylpenicillin, ceftriaxone, and sulfamethoxazole–trimethoprim were among the most frequently used agents in combination therapy. The temporal distribution of antibiotics prescribed in combination therapy is presented in Table 2. A marked temporal variation was observed, with 82 prescriptions in 2019, a substantial reduction to 23 in 2020, and a subsequent increase to 116 in 2021, totaling 221 antibiotic prescriptions involving combination therapy over the three-year period. Azithromycin showed the highest number of prescriptions in combination therapy (*n* = 54), followed by ceftriaxone (*n* = 25) and benzathine benzylpenicillin (*n* = 23).

**Table 2:** Number of antibiotics prescribed in combination therapy by year.

Antibiotic	2019, n (%)	2020, n (%)	2021, n (%)	Total, n (%)
Amoxicillin	17 (20.7)	2 (8.7)	12 (10.3)	31 (14.0)
Amoxicillin–clavulanate	3 (3.7)	0 (0.0)	5 (4.3)	8 (3.6)
Azithromycin	5 (6.1)	9 (39.1)	40 (34.5)	54 (24.4)
Benzathine benzylpenicillin	14 (17.1)	2 (8.7)	7 (6.0)	23 (10.4)
Cephalexin	12 (14.6)	0 (0.0)	4 (3.4)	16 (7.2)
Ceftriaxone	4 (4.9)	5 (21.7)	16 (13.8)	25 (11.3)
Others	27 (32.9)	5 (21.7)	32 (27.6)	64 (29.0)
Total	82 (100.0)	23 (100.0)	116 (100.0)	221 (100.0)

Percentages are based on annual totals; overall percentages are based on all combination-therapy prescriptions (*n* = 221).

### 3.3 Direct antibiotic acquisition costs

The estimated minimum cumulative direct antibiotic acquisition cost for the 10,489 prescription records during the study period was USD 13,044.67. Annual expenditure was highest in 2019 (USD 6263.85), decreased in 2020 (USD 2480.02), and increased again in 2021 (USD 4300.80). The highest cumulative costs were associated with benzathine benzylpenicillin (USD 5604.21), followed by ceftriaxone (USD 2160.65), amoxicillin (USD 2021.71), and azithromycin (USD 1195.09).

For antibiotics prescribed as part of combination therapy, the estimated minimum direct acquisition cost was USD 189.36, with the highest annual expenditure observed in 2021 (USD 93.22). Annual direct antibiotic acquisition costs are summarized in Table 3.

**Table 3:** Estimated minimum direct antibiotic acquisition costs by year (nominal USD).

Variable	2019 (USD)	2020 (USD)	2021 (USD)	Total (USD)
Amoxicillin	1227.37	283.80	510.55	2021.71
Azithromycin	168.36	448.56	578.16	1195.09
Benzathine benzylpenicillin	2845.63	1049.89	1708.69	5604.21
Ceftriaxone	824.82	343.19	992.64	2160.65
Overall estimated minimum direct cost	6263.85	2480.02	4300.80	13,044.67
Combination therapies	72.77	23.37	93.22	189.36

USD, United States dollars. Costs are expressed as nominal values and represent minimum estimated direct antibiotic acquisition costs based on unit acquisition prices and prescription frequencies, assuming one acquisition unit per prescription; treatment duration and dosing were not considered.

### 3.4 Antibiotic unit-cost variation according to diagnosis and hospital-transfer status

Among the 324 treatment episodes included in the cost analysis, the mean antibiotic unit cost was USD 1.31 (SD = 0.39), with a median of USD 1.54 (IQR = 0.49) and values ranging from USD 0.25 to USD 2.07. Visual inspection of the histogram and Q–Q plot indicated departures from normality, which were confirmed by the Shapiro–Wilk test (W = 0.893, *p* < 0.001). Residuals from the preliminary ANOVA model were also non-normally distributed (W = 0.944, *p* < 0.001), whereas Levene's test did not indicate heterogeneity of variances across diagnostic groups [F(6,317) = 0.896, *p* = 0.498]. Therefore, non-parametric tests were used for the primary inferential analyses. Detailed statistical results and diagnostic plots are provided in the Supplementary Data (Statistical_Diagnostics worksheet; Tables S1–S5 and Figs. S1–S3).

Antibiotic unit costs differed significantly across aggregated diagnostic groups [Kruskal–Wallis χ^2^(6) = 14.785, *p* = 0.022]. However, Dunn's post-hoc comparisons with Holm adjustment identified no statistically significant pairwise differences (all adjusted *p* > 0.05). Descriptive unit-cost distributions across diagnostic groups are presented in Table 4.

**Table 4:** Antibiotic unit costs according to aggregated diagnostic group.

Diagnostic group	n	Median (USD)	IQR (USD)	Range (USD)
Fever syndrome	18	1.54	0.39	0.80–2.07
Other	85	1.50	0.39	0.25–2.07
Otitis	7	1.15	0.05	0.80–2.07
Respiratory infection	38	1.15	0.78	0.80–2.07
Skin infection	28	1.54	0.39	1.15–2.02
Tonsillitis/Pharyngitis	145	1.54	0.74	0.25–2.02
UTI	3	1.59	0.24	1.59–2.07

IQR, interquartile range; UTI, urinary tract infection; USD, United States dollars. Overall comparison: Kruskal–Wallis *χ*^2^(6) = 14.785, *p* = 0.022. Dunn's post hoc test with Holm adjustment identified no statistically significant pairwise differences (all adjusted *p* > 0.05).

In an exploratory comparison, antibiotic unit costs also differed according to hospital-transfer status. Transferred episodes had a median unit cost of USD 1.59 (IQR = 0.00), compared with USD 1.15 (IQR = 0.74) among non-transferred episodes (Wilcoxon rank-sum test, W = 161, *p* < 0.001) (Table 5). The estimated location shift for non-transferred versus transferred episodes was −USD 0.44 (95% confidence interval (CI): −0.44 to −0.04). However, only seven episodes involved hospital transfer, all of which had the same recorded unit cost. Given this small and highly imbalanced group, the finding should be considered exploratory and should not be interpreted as evidence that higher antibiotic expenditure improves clinical outcomes.

**Table 5:** Antibiotic unit costs according to hospital-transfer status.

Hospital-transfer status	n (%)	Mean ± SD (USD)	Median (IQR), USD	Range (USD)
Transferred	7 (2.3)	1.59 ± 0.00	1.59 (0.00)	1.59–1.59
Non-transferred	295 (97.7)	1.26 ± 0.35	1.15 (0.74)	0.25–2.07

IQR, interquartile range; SD, standard deviation; USD, United States dollars. Percentages were calculated using the hospital-transfer analysis sample (*n* = 302). Wilcoxon rank-sum test: *W* = 161, *p* < 0.001; estimated location shift for non-transferred versus transferred episodes = −USD 0.44 (95% CI: −0.44 to − 0.04).

### 3.5 Exploratory multivariate analysis of therapeutic profiles

Exploratory HCA and PCA were performed to characterize similarities and differences in therapeutic profiles across the seven diagnostic groups (Fig. 2A–B). The HCA provided a complementary assessment of similarities among diagnostic groups (Fig. 2A), whereas the PCA summarized the main dimensions of variation and the therapeutic variables contributing to these patterns (Fig. 2B).

**Fig. 2:**
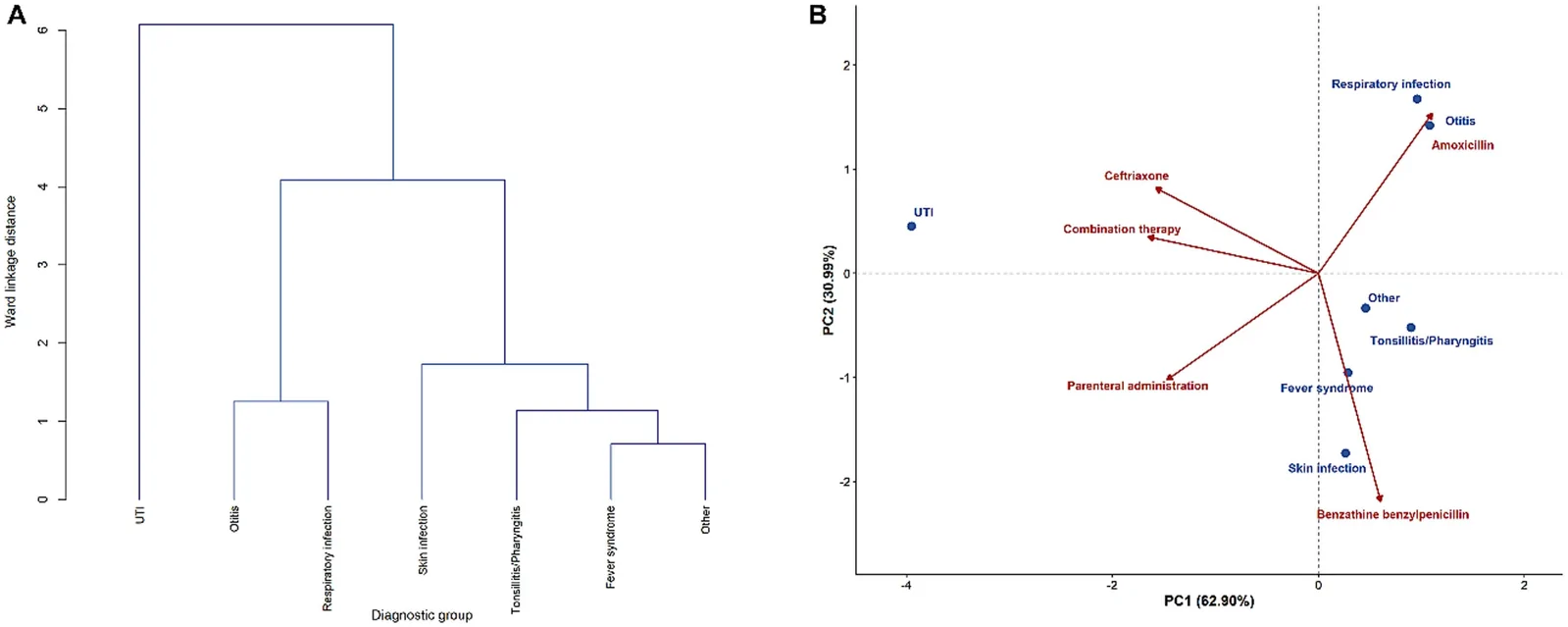
Exploratory multivariate analysis of therapeutic profiles across diagnostic groups. (A) Hierarchical cluster analysis (HCA) dendrogram based on five standardized therapeutic indicators—parenteral administration, combination therapy, amoxicillin, benzathine benzylpenicillin, and ceftriaxone—using Euclidean distance and Ward's linkage method. (B) Principal component analysis (PCA) biplot based on the same standardized variables. The first two principal components explained 93.89% of the total variance (PC1 = 62.90%; PC2 = 30.99%). Blue points represent diagnostic groups, and red vectors represent variable loadings. Both analyses included seven aggregated diagnostic groups. (For interpretation of the references to colour in this figure legend, the reader is referred to the web version of this article.)

The HCA showed a marked dissimilarity of the UTI therapeutic profile relative to the other diagnostic groups. Among the remaining diagnoses, closer similarities were observed between Otitis and Respiratory infection and between Fever syndrome and Other. These clustering patterns illustrated similarities and differences in therapeutic management across diagnostic groups.

The first two principal components of the PCA explained 93.89% of the total variance, with the first principal component (PC1) accounting for 62.90% and the second principal component (PC2) for 30.99%. UTI showed a marked separation from the remaining diagnostic groups, primarily along PC1, and was positioned in the direction of parenteral administration and combination therapy, suggesting that these therapeutic characteristics contributed to its distinct multivariate profile. In contrast, Respiratory infection and Otitis were located on the opposite side of PC1 and showed similar profiles, with their positions more closely aligned with amoxicillin use. Along PC2, benzathine benzylpenicillin showed a strong negative loading, contributing to the positioning of diagnostic groups such as Tonsillitis/Pharyngitis and Fever syndrome in the lower portion of the biplot. Ceftriaxone, combination therapy, and parenteral administration contributed in different directions to the separation of the remaining diagnostic profiles. Complete PCA loading values are provided in the Supplementary Data (PCA_HCA_Analysis worksheet; Table S6).

Overall, the HCA patterns were broadly consistent with the relative positioning of the diagnostic groups in the PCA, providing complementary descriptive representations of the therapeutic profiles. The aggregated therapeutic profiles used for both PCA and HCA are provided in the Supplementary Data (PCA_HCA_Analysis worksheet; Table S7). Given the aggregation of the data at the diagnostic-group level and the small number of UTI episodes, these multivariate findings should be interpreted as exploratory.

### 3.6 WHO AWaRe classification

According to the WHO AWaRe classification, Access antibiotics accounted for 78.1% of the 324 treatment episodes included in the cost analysis (*n* = 253), whereas Watch antibiotics accounted for 21.9% (*n* = 71). No Reserve antibiotics were identified. The median antibiotic unit cost was USD 1.50 (IQR = 0.74) for Access antibiotics and USD 1.59 (IQR = 0.44) for Watch antibiotics (Table 6).

**Table 6:** WHO AWaRe classification of prescribed antimicrobials.

AWaRe category	n (%)	Mean ± SD (USD)	Median (IQR), USD	Min–Max (USD)
Access	253 (78.1)	1.27 ± 0.40	1.50 (0.74)	0.25–2.02
Watch	71 (21.9)	1.44 ± 0.29	1.59 (0.44)	1.15–2.07

**Note:** AWaRe, Access, Watch, and Reserve; IQR, interquartile range; SD, standard deviation; USD, United States dollars. No Reserve antibiotics were identified.

## 4 Discussion

The present study provides evidence on antibiotic prescribing patterns and direct antibiotic acquisition costs in a pediatric emergency care unit in Northeastern Brazil. The findings indicate a substantial concentration of antibiotic use among children aged 0–2 years, which is consistent with the increased susceptibility of this age group to acute infectious conditions, particularly respiratory and febrile illnesses.^14^ This age-related concentration reinforces the importance of implementing age-specific prescribing protocols and antimicrobial stewardship strategies in pediatric urgent care services.15., 16.

A relevant finding of this study was the marked temporal variation in antibiotic prescribing and associated costs across the study period. A reduction in prescriptions and direct costs was observed in 2020, followed by a marked increase in 2021. This fluctuation coincided with the COVID-19 pandemic period and may reflect several concurrent factors, including reduced healthcare utilization, changes in healthcare-seeking behavior, altered patterns of pediatric respiratory infections associated with infection-control measures, and changes in clinical practice.^17^ However, given the retrospective design and the absence of data specifically addressing these mechanisms, the present study cannot determine the factors responsible for the observed temporal variation. Similar shifts in antimicrobial consumption have been reported in emergency and hospital settings during periods of epidemiological transition.18., 19.

Among the prescribed agents, azithromycin, benzathine benzylpenicillin, ceftriaxone, and amoxicillin accounted for the highest prescription frequencies and cumulative costs. The predominance of these antibiotics likely reflects their routine use in the management of common pediatric infections, including tonsillitis, suspected bacterial respiratory infections, and febrile syndromes. However, the frequent use of azithromycin deserves particular attention, as macrolides are generally recommended as alternative agents, particularly in cases of β-lactam allergy.20., 21. Nevertheless, prescribing frequency alone does not establish inappropriate use. Assessment of prescribing appropriateness would require additional information on clinical indication, β-lactam allergy, local resistance patterns, guideline concordance, and the availability of narrower-spectrum alternatives. Therefore, the observed pattern warrants further prescribing audit and antimicrobial stewardship assessment.22., 23.

The AWaRe analysis provided an additional antimicrobial stewardship perspective. Among the 324 treatment episodes included in the cost analysis, Access antibiotics accounted for 78.1% (*n* = 253), whereas Watch antibiotics accounted for 21.9% (*n* = 71), with no Reserve antibiotics identified. Although the predominance of Access agents is relevant from a stewardship perspective, the proportion of Watch antibiotics highlights the importance of continued monitoring of local prescribing practices. Importantly, AWaRe categorization is descriptive and does not, by itself, establish the clinical appropriateness of individual prescriptions. These findings also reinforce the need to monitor Watch antibiotic use because of its potential implications for antimicrobial resistance. Targeted stewardship interventions, including local prescribing protocols and periodic prescription review, may help optimize empirical antibiotic use in pediatric emergency care.

The analysis of direct antibiotic acquisition costs showed that benzathine benzylpenicillin and ceftriaxone accounted for the highest cumulative expenditures during the study period. Although antibiotic unit costs remained relatively low, the estimated minimum cumulative acquisition cost was substantial, particularly considering the high volume of pediatric emergency visits. From a health economics perspective, these findings reinforce that even low unit-cost medications may generate a considerable financial burden when prescribed at scale in public healthcare services.8., 24.

An important result of this study was the statistically significant overall variation in antibiotic unit costs across aggregated diagnostic groups. However, post hoc comparisons did not identify significant pairwise differences after adjustment for multiple testing. This finding suggests heterogeneity in antibiotic cost profiles across diagnostic categories, although the specific groups driving this variation could not be established conclusively. Such differences may reflect variation in prescribing patterns, route of administration, and the use of different antimicrobial agents across diagnostic groups.25., 26., 27.

The exploratory multivariate analyses further highlighted differences in therapeutic profiles across diagnostic groups (Fig. 2). HCA identified UTI as the most dissimilar therapeutic profile, while closer similarities were observed between otitis and respiratory infection and between fever syndrome and other diagnoses. Consistent with these patterns, the PCA showed a marked separation of UTI, positioned in the direction of parenteral administration, combination therapy, and ceftriaxone, whereas respiratory infection and otitis showed more similar profiles and were positioned in the direction of amoxicillin use. Benzathine benzylpenicillin contributed strongly to the negative direction of PC2, along which skin infection, fever syndrome, and tonsillitis/pharyngitis were positioned. Overall, the complementary HCA and PCA findings illustrate differences in antibiotic selection and administration across diagnostic groups and should be interpreted as exploratory, descriptive patterns at the aggregated diagnostic-group level.

The cost comparison according to hospital-transfer status showed that higher antibiotic unit costs were observed among transferred episodes compared with non-transferred episodes. This observed difference should be interpreted as an exploratory finding rather than evidence of a robust association between antibiotic unit costs and hospital-transfer status or of greater treatment effectiveness. The transferred group was considerably smaller (*n* = 7 vs. *n* = 295 non-transferred episodes), resulting in a marked imbalance between groups that limits the statistical robustness of this comparison and increases its susceptibility to random error. In addition, hospital-transfer status represents an intermediate outcome that may be influenced by factors beyond clinical response, including institutional capacity, physician decision-making, social circumstances, diagnostic uncertainty, and referral protocols. Therefore, the observed difference should be interpreted cautiously and does not establish a causal relationship between higher antibiotic unit costs and improved clinical outcomes.

This study has relevant implications for clinical practice and health policy. The findings support antimicrobial stewardship strategies focused on pediatric emergency care and may inform prescribing protocols and formulary management within the *SUS*. Integrating prescribing and cost surveillance may also help identify opportunities for cost optimization while maintaining clinical appropriateness as the primary consideration.7., 27., 28., 29.

Some limitations should be acknowledged. First, the retrospective design limited control over data completeness and variable standardization and did not allow a comprehensive assessment of antibiotic indication appropriateness, dosing, treatment duration, or adherence to clinical guidelines. Therefore, the AWaRe classification should be interpreted as a descriptive characterization of prescribing patterns rather than as an assessment of antimicrobial appropriateness. Second, the use of direct antibiotic acquisition costs alone does not capture the full economic burden of pediatric emergency care. Third, hospital transfer was used as an intermediate outcome and should not be interpreted as a direct measure of clinical cure or antibiotic effectiveness. Fourth, disease severity was not systematically measured or adjusted for and may represent an important source of confounding by indication. Microbiological confirmation was not consistently available, and potential diagnostic or coding errors inherent to retrospective medical records cannot be excluded.

In addition, missing outcome data and the substantial reduction from 10,489 prescription records to 324 treatment episodes for antibiotic unit-cost analysis and 302 episodes for the hospital-transfer analysis may have introduced selection bias and limited the representativeness of the analytical samples. Referral patterns may also have introduced bias in the hospital-transfer analysis. Given the observational design and potential residual confounding, causal relationships cannot be inferred. As this was a single-center study conducted in Camaçari, Bahia, the findings primarily reflect local prescribing practices and healthcare organization. Although they may be relevant to SUS emergency care units with similar characteristics, extrapolation to other healthcare settings and regions should be made with caution. Despite these limitations, the study provides relevant real-world evidence on antibiotic use and direct antibiotic acquisition costs in an understudied pediatric emergency setting in Brazil.

## 5 Practical implications

The findings of this study have relevant practical implications for pediatric emergency care and public health management. The high frequency of antibiotic prescribing among children aged 0–2 years and the estimated minimum cumulative direct antibiotic acquisition costs highlight opportunities to strengthen antimicrobial stewardship in urgent care settings. Periodic review of antibiotic selection, particularly Watch agents, and the development of locally adapted prescribing protocols may contribute to more informed antimicrobial management.

The observed variation in antibiotic unit costs across diagnostic groups highlights the potential value of strengthening diagnostic and prescribing pathways in pediatric emergency care. Although the overall difference across diagnostic groups was statistically significant, no specific pairwise differences remained significant after adjustment for multiple comparisons. Therefore, these findings support continued evaluation of diagnosis-specific prescribing patterns rather than targeting any single diagnostic group.

Furthermore, monitoring antibiotic acquisition expenditures alongside prescribing patterns may support formulary planning, procurement strategies, and protocol standardization within the SUS. However, the available data do not establish inappropriate prescribing or demonstrate reductions in antimicrobial resistance or total healthcare costs; these potential benefits require prospective evaluation.

## 6 Conclusion

This study demonstrated that antibiotic prescribing in a pediatric emergency care unit was associated with estimated minimum direct antibiotic acquisition costs. The highest estimated cumulative acquisition costs were associated with benzathine benzylpenicillin, ceftriaxone, amoxicillin, and azithromycin, reflecting their frequent use in common pediatric infectious presentations.

Antibiotic unit costs varied significantly across diagnostic groups, although no specific pairwise differences remained significant after adjustment for multiple comparisons. Exploratory multivariate analyses further highlighted distinct therapeutic profiles across diagnostic groups, with urinary tract infections showing a distinct profile characterized by greater alignment with parenteral administration, combination therapy, and ceftriaxone.

Overall, these findings reinforce the importance of antimicrobial stewardship strategies, protocol standardization, and evidence-based resource planning in pediatric urgent care services, particularly within the SUS.

## Declaration of use of AI-assisted technologies

During the preparation of this work, the authors used artificial intelligence-assisted language tools to improve grammar, readability, and clarity. The authors reviewed and edited the content as needed and take full responsibility for the final content of the publication.

## CRediT authorship contribution statement

**Cristiane Almeida Rocha:** Writing – review & editing, Writing – original draft, Visualization, Validation, Methodology, Investigation, Formal analysis, Data curation, Conceptualization. **Eduard F. Valenzuela:** Writing – review & editing, Writing – original draft, Visualization, Validation, Software, Methodology, Investigation, Formal analysis. **Fernanda Warken Rosa:** Writing – review & editing, Writing – original draft, Visualization, Validation, Supervision, Methodology, Investigation. **Aníbal de Freitas Santos Júnior:** Writing – review & editing, Writing – original draft, Visualization, Validation, Supervision, Methodology, Investigation. **Roberto Rodrigues Bandeira Tosta Maciel:** Writing – review & editing, Writing – original draft, Visualization, Validation, Supervision, Project administration, Methodology, Investigation, Data curation, Conceptualization.

## Ethical approval

This study was approved by the Research Ethics Committee (*Comitê de Ética em Pesquisa – CEP*) of the *Universidade do Estado da Bahia (UNEB)*, Brazil, on August 7, 2021, under approval number 4.891.546 (CAAE: 48337621.1.0000.0057). The committee approved the waiver of informed consent and assent, given the retrospective use of medical records. Patient confidentiality and anonymity were maintained throughout the study.

## Code availability

The R scripts used for statistical analyses and data visualization are available from the corresponding author upon reasonable request.

## Declaration of competing interest

The authors declare that they have no known competing financial interests or personal relationships that could have appeared to influence the work reported in this paper.

## Data Availability

The patient-level datasets generated and/or analyzed during this study are not publicly available because they contain information derived from pediatric clinical records and are subject to institutional and ethical restrictions. The analytical dataset used for the antibiotic cost analyses is provided in the Supplementary Data. Additional de-identified analytical data and supporting materials may be made available upon reasonable request to the corresponding author, subject to institutional authorization and applicable data-protection requirements. The availability of additional underlying clinical records cannot be guaranteed.
